# Single-molecule analysis of subtelomeres and telomeres in Alternative Lengthening of Telomeres (ALT) cells

**DOI:** 10.1186/s12864-020-06901-7

**Published:** 2020-07-15

**Authors:** Heba Z. Abid, Jennifer McCaffrey, Kaitlin Raseley, Eleanor Young, Katy Lassahn, Dharma Varapula, Harold Riethman, Ming Xiao

**Affiliations:** 1 School of Biomedical Engineering, Science and Health Systems, Drexel University, Philadelphia, PA USA; 2 School of Medical Diagnostic and Transnational Sciences, Old Dominion University, Norfolk, VA USA; 3 Institute of Molecular Medicine and Infectious Disease, School of Medicine, Drexel University, Philadelphia, PA USA

**Keywords:** Genomics, Cancer telomeres, Alternative lengthening of telomeres (ALT), U2OS, SK-MEL-2, Saos-2, Single molecule optical mapping

## Abstract

**Background:**

Telomeric DNA is typically comprised of G-rich tandem repeat motifs and maintained by telomerase (Greider CW, Blackburn EH; Cell 51:887–898; 1987). In eukaryotes lacking telomerase, a variety of DNA repair and DNA recombination based pathways for telomere maintenance have evolved in organisms normally dependent upon telomerase for telomere elongation (Webb CJ, Wu Y, Zakian VA; Cold Spring Harb Perspect Biol 5:a012666; 2013); collectively called Alternative Lengthening of Telomeres (ALT) pathways. By measuring (TTAGGG) n tract lengths from the same large DNA molecules that were optically mapped, we simultaneously analyzed telomere length dynamics and subtelomere-linked structural changes at a large number of specific subtelomeric loci in the ALT-positive cell lines U2OS, SK-MEL-2 and Saos-2.

**Results:**

Our results revealed loci-specific ALT telomere features. For example, while each subtelomere included examples of single molecules with terminal (TTAGGG) n tracts as well as examples of recombinant telomeric single molecules, the ratio of these molecules was subtelomere-specific, ranging from 33:1 (19p) to 1:25 (19q) in U2OS. The Saos-2 cell line shows a similar percentage of recombinant telomeres. The frequency of recombinant subtelomeres of SK-MEL-2 (11%) is about half that of U2OS and Saos-2 (24 and 19% respectively). Terminal (TTAGGG) n tract lengths and heterogeneity levels, the frequencies of telomere signal-free ends, and the frequency and size of retained internal telomere-like sequences (ITSs) at recombinant telomere fusion junctions all varied according to the specific subtelomere involved in a particular cell line. Very large linear extrachromosomal telomere repeat (ECTR) DNA molecules were found in all three cell lines; these are in principle capable of templating synthesis of new long telomere tracts via break-induced repair (BIR) long-tract DNA synthesis mechanisms and contributing to the very long telomere tract length and heterogeneity characteristic of ALT cells. Many of longest telomere tracts (both end-telomeres and linear ECTRs) displayed punctate CRISPR/Cas9-dependent (TTAGGG) n labeling patterns indicative of interspersion of stretches of non-canonical telomere repeats.

**Conclusion:**

Identifying individual subtelomeres and characterizing linked telomere (TTAGGG) n tract lengths and structural changes using our new single-molecule methodologies reveals the structural consequences of telomere damage, repair and recombination mechanisms in human ALT cells in unprecedented molecular detail and significant differences in different ALT-positive cell lines.

## Background

Telomeres are nucleoprotein structures located at the tips of eukaryotic chromosomes that prevent the ends of the linear DNA component of chromosomes from being recognized and processed as double-strand breaks, and which provide a means for the faithful completion of chromosomal DNA replication [1, 2]. Telomeric DNA is typically comprised of G-rich tandem repeat motifs; the precise sequence of the telomeric DNA motif is determined by the species-dependent RNA component of the RNP enzyme telomerase. Telomerase adds DNA copies of this motif to existing telomeric DNA at chromosome ends [3]. In eukaryotes lacking telomerase, telomeres can be maintained via the activity of retrotransposons [4] and in some cases by epigenetically regulated protection of DNA ends not ordinarily considered telomeric [5, 6]. A variety of DNA repair and DNA recombination based pathways for telomere maintenance have evolved in organisms normally dependent upon telomerase for telomere elongation [1]; collectively called Alternative Lengthening of Telomeres (ALT) pathways, they can become activated or up-regulated in the absence of telomerase activity.

Human telomeric DNA is comprised of 5’TTAGGG3’ motifs [7]. Most human somatic tissues do not contain an active telomere maintenance mechanism, which results in the loss of telomere repeats with each somatic cell division due to the “end replication problem” as well as telomeric DNA end processing [7, 8]. The telomere nucleoprotein structure breaks down when telomeric DNA tracts become critically short, causing telomere dysfunction-mediated senescence or apoptosis [9–12] or, in the absence of functional DNA Damage Response (DDR) checkpoint pathways, aberrant telomeric DNA repair, telomere fusions, and ongoing genome instability [13]. In human cancer cells, telomere maintenance pathways have become re-activated, stabilizing the cancer genome and enabling unlimited cellular proliferation. While most cancers have an activated telomerase pathway for maintaining telomeres [14], a significant number (about 10–15%) lack telomerase and maintain their telomeres using ALT mechanisms [8].

ALT is most prevalent in specific cancer types, including osteosarcoma and glioblastoma and are usually associated with a poor prognosis [8, 15]. Human ALT has long been hypothesized to involve double strand break induced homologous recombination (HR) mechanisms [16]. This is supported by evidence that genes encoding HR proteins are necessary for telomere-length maintenance in human ALT cells. Also, circumstantial evidence has been provided in ALT cells that many HR proteins are present with telomeric DNA and telomere-binding proteins in promyelocytic leukemia (PML) bodies called ALT-associated PML bodies (APBs), where multiple ALT telomeres can cluster and exchange DNA via HR-dependent mechanisms [8, 15]. ALT probably includes strand invasion of the template molecule and formation of an HR intermediate structure [8, 17]. Several phenotypic characteristics of ALT cells differentiate them from telomerase-positive cancer cells. ALT cells have highly heterogeneous chromosomal telomere lengths that range from undetectable to extremely long and these lengths can rapidly change [8]. Visualization of telomeres in ALT cell populations with fluorescence in situ hybridization (FISH) has confirmed this characteristic. One study showed that within one cell, some chromosome ends had no detectable telomeric sequences while others had very strong telomere signals indicating extremely long telomere tracts [18] or spatially clustered telomeres prone to homologous recombination [19, 20]. Associated with greatly elevated levels of recombination at telomeres in ALT cells are an abundance of telomeric DNA that is separate from chromosomes. The extrachromosomal telomeric DNA is either double-stranded telomeric circles (t-circles), single- stranded circles (either C-circles or G-circles depending on the DNA strand of origin), linear double-stranded DNA, or “t-complex” DNA that is most likely a highly branched structure [8].

Recent work suggests that ALT is a highly regulated telomere repair pathway [21]. Telomere DNA damage caused by TERRA transcription-induced R-loops within (TTAGGG) n tracts [22, 23], dysfunctional ATRX [24, 25], and replication stalling at telomeres [26] initiates DS break-dependent homology directed repair (Break-induced Repair (BIR)) synthesis of long telomere tracts [27, 28]; this telomere lengthening repair mechanism is counteracted by BLM/SLX-mediated HR processing steps [21] and active telomere trimming mediated by TZAP [29, 30]. Multiple non-canonical as well as classical DNA repair pathways appear to be active at ALT telomeres [31]. In order to help decipher these mechanisms and their consequences for ALT cancers, it is critical to characterize the telomere-associated DNA structures at ALT telomeres in these cells at the highest resolution possible. We have utilized our recently-developed single-molecule method that simultaneously measures individual telomere (TTAGGG) n tract lengths and identifies their physically linked DNA to analyze these structures in the telomerase-negative ALT-positive U2OS human osteosarcoma cancer cell line, SK-MEL-2 melanoma cell line and Saos-2 osteosarcoma *cell* line. We describe patterns of telomere (TTAGGG) n tracts associated with specific subtelomeres, revealing multiple types of telomeric DNA structures associated with DNA repair events in ALT-positive cells and providing unique insights into ALT.

## Results

Our recently developed two-color labeling scheme was performed on U2OS, SK-MEL-2 and Saos-2 genomic DNA to acquire global subtelomere-specific single-telomere lengths [32] and associated subtelomere-specific single-molecule structural data. Telomere (TTAGGG) n tracts were specifically labeled with fluorescent dyes by CRISPR-Cas9 nick labeling. The telomere labeling intensity is used to estimate the telomere (TTAGGG) n tract length [32]. Simultaneously, the genomic DNA is globally nick-labeled using Nt.BspQI to target the GCTCTTC motif. The labeled DNA molecules are then optically imaged in a high-throughput manner using nanochannel arrays [33]. De novo assembly of optically mapped, large single DNA fragments of genomic DNA is performed and unique Nt.BspQI patterns are used to map assemblies to subtelomeric reference sequences (human hg38), which allows for identification of the specific subtelomeres, quantitation of the linked single telomere (TTAGGG) n tract lengths, and detection of recombinant single molecules containing intact subtelomeres [34–36].

Globally, we measured and analyzed an average of 30 out of 46 subtelomeres with approximately 30 molecules per chromosome arm (Tables 1, 2 and 3). The chromosome arms 13p, 14p, 15p, 21p, 22p, XpYp could not be identified and measured due to the lack of reference sequences or many gaps in the reference (indicated as nr in Tables 1, 2 and 3). Chromosome arms 16p, 17p, and 22q subtelomeres failed the assembly with most samples because of inverted nick pair (INP) sites in the subtelomere [35] . These are two closely spaced nicking enzyme sites on opposite strands which causes double-stranded breaks in molecules to be mapped, precluding their assembly and localization to the reference sequence. Several subtelomeres (4q, 10p, 10q and XqYq in U2OS 4q; 5p, and 11p in SK-MEL; 2q, 7p, 8p, 9q, 11q, 12q, 15q, 20q and XqYq in Saos-2 indicated as N/A in Tables 1, 2 and 3) did not have enough assembled molecules for analysis; we believe the most likely explanation for this are high levels of recombination within these subtelomeres that would interfere with assembly of consensus maps, although there are other possible explanations (see discussion). The linked telomere (TTAGGG) n tract length data and subtelomere-associated structural data for each of these subtelomeres is summarized in Tables 1, 2 and 3.

**Table 1:** U2OS telomere lengths

	End telomere	End telomere loss	Recombined Ends with ITS	ITS Loss	(TTAGGG) *n* < 500 bp	% Recombinant Subtelomere Molecules of total analyzed for subtelomere	Longest Telomere
Chr-parm	Mean Length (kb) ± Std (# telomeres)	(# molecules)	Mean Length (kb) ± Std (# telomeres)	(# molecules)	# molecules (# end, # ITS)		Length (kb)
1p	3.8 ± 4.4 (15)	1	4.4 ± 3.6 (4)	5	4 (3,1)	36	17.1
2p	2.1 ± 2.5 (12)	2	4.3 ± 4.7 (4)	2	5 (4,1)	30	10.7
3p	6.6 ± 8.4 (16)	0	2.9 ± 3.8 (7)	0	5 (1,4)	30	35.8
4p	3.2 ± 3.4 (19)	0	1.9 ± 2.1 (2)	1	7 (6,1)	14	11.8
5p	3.7 ± 4.2 (21)	3	0.1 ± 0.1 (9)	3	10 (1,9)	33	15.5
6p	2.8 ± 1.8 (8)	0	0.6 ± 1.1 (6)	0	6 (1,5)	43	6
7p	3.4 ± 7.0 (5)	0	0.5 ± 0.4 (17)	0	12 (3,9)	77	15.8
8p	2.7 ± 3.3 (8)	0	0.2 ± 0.2 (3)	0	6 (3,3)	28	8.4
9p	2.6 ± 4.9 (7)	2	4.7 ± 7.4 (5)	3	3 (2,1)	47	17.8
10p	N/A	N/A	N/A	N/A	N/A	N/A	N/A
11p	4.4 ± 5.7 (12)	0	0.7 ± 0.1 (3)	10	1 (1,0)	52	18.1
12p	3.8 ± 3.3 (20)	2	27.6 ± 0 (1)	11	2 (2,0)	35	27.6
13p	nr		nr				
14p	nr		nr				
15p	nr		nr				
16p	INP		INP				
17p	INP		INP				
18p	3.7 ± 5.1 (10)	1	0	3	1 (1,0)	21	16.6
19p	7.0 ± 8.1 (29)	1	3.2 ± 0 (1)	0	1 (1,0)	3	35.9
20p	7.8 ± 12.2 (20)	0	3.5 ± 0.6 (2)	1	2 (2,0)	13	47.5
21p	nr		nr				
22p	nr		nr				
Xp/Yp	nr		nr				
Chr-qarm							
1q	3.4 ± 3.9 (6)	1	0.6 ± 0.6 (18)	4	8 (1,7)	76	9.4
2q	5.5 ± 6.1 (30)	0	1.1 ± 0 (1)	2	5 (5,0)	9	23.8
3q	0 (7)	7	0	16	0	70	N/A
4q	N/A	N/A	N/A	N/A	N/A	N/A	N/A
5q	5.5 ± 5.6 (30)	0	6.6 ± 3.6 (3)	0	2 (2,0)	9	24.2
6q	7.8 ± 7.9 (21)	0	4.8 ± 1.5 (2)	0	2 (2,0)	9	28.2
7q	4.9 ± 4.4 (11)	0	7.9 ± 5.4 (2)	0	1 (1,0)	15	11.7
8q	1.8 ± 3.5 (23)	6	0.4 ± 0.3 (19)	7	15 (6,9)	52	12.9
9q	5.2 ± 5.3 (23)	0	3.3 ± 2.5 (6)	2	1 (1,0)	26	17.7
10q	3.8 ± 6.4 (2)	0	0	2	0	50	13.4
11q	4.2 ± 3.1 (7)	1	0.2 ± 0.2 (4)	1	4 (0,4)	38	8.5
12q	4.1 ± 4.7 (18)	4	0.7 ± 1.0 (7)	3	7 (3,4)	31	16.5
13q	5.5 ± 5.4 (30)	0	8.6 ± 5.8 (2)	0	1 (1,0)	6	20.9
14q	2.1 ± 4.8 (6)	10	0	4	0	20	16.7
15q	4.7 ± 5.7 (17)	6	0.4 ± 0.5 (3)	4	6 (4,2)	23	18.5
16q	5.3 ± 5.3 (28)	4	4.5 ± 4.2 (7)	0	4 (2,2)	18	17.1
17q	5.4 ± 5.3 (30)	0	6.0 ± 5.9 (2)	3	4 (4,0)	14	18.2
18q	0.5 ± 1.2 (5)	4	0.9 ± 0.7 (26)	5	14 (2,12)	78	2.9
19q	nd	1	1.5 ± 1.1 (25)	0	4 (0,4)	96	5
20q	3.4 ± 3.9 (21)	0	1.3 ± 1.1 (3)	0	6 (5,1)	13	14.2
21q	4.9 ± 4.7 (12)	1	1.9 ± 4.0 (13)	17	9 (1,8)	70	16.2
22q	INP		INP				
Xq/Yq	N/A	N/A	N/A	N/A	N/A	N/A	N/A
total	724	57	182	108	158 (65,93)	24

**Table 2:** SK-MEL-2 telomere lengths

	End telomere	End telomere loss	Recombined Ends with ITS	ITS Loss	(TTAGGG) n < 500 bp	% Recombinant Subtelomere Molecules % of total analyzed for subtelomere	Longest Telomere
Chr-parm	Mean Length (kb) ± Std (# telomeres)	(# molecules)	Mean Length (kb) ± Std (# telomeres)	(# molecules)	# molecules (# end, # ITS)		Length (kb)
1p	4.5 ± 3.4 (24)	0	nd	0	1 (1,0)	0	12.2
2p	3.1 ± 4.1 (33)	2	nd	0	5 (5,0)	0	18.8
3p	4.2. ± 3.6 (28)	0	nd	0	5 (4,0)	0	14.6
4p	2.9 ± 3.2 (4)	0	nd	0	0 (0,0)	0	7.7
5p	N/A	N/A	N/A	N/A	N/A	N/A	N/A
6p	2.1 ± 2.1 (13)	0	nd	0	1 (1,0)	0	7.5
7p	1.8 ± 2.0 (14)	2	nd	0	3 (3,0)	0	5.7
8p	2.9 ± 2.8 (31)	5	10.3 ± 0 (1)	0	5 (5,0)	3	11.7
9p	3.1 ± 3.2 (34)	1	2.6 ± 2.5 (5)	0	4 (4,0)	13	14.1
10p	3.2 ± 3.2 (26)	2	2.9 ± 0 (1)	0	2 (2,0)	4	11.2
11p	N/A	N/A	N/A	N/A	N/A	N/A	N/A
12p	2.6 ± 2.0 (27)	2	4.0 ± 4.5 (2)	0	2 (2,0)	7	6.6
13p	nr		nr				
14p	nr		nr				
15p	nr		nr				
16p	INP		INP				
17p	INP		INP				
18p	2.2 ± 2.6 (9)	1	1.1 ± 0 (1)	0	1 (1,0)	10	8.7
19p	2.7 ± 3.0 (32)	1	5.1 ± 3.9 (5)	0	4 (4,0)	14	13.2
20p	4.8 ± 5.2 (16)	1	4.6 ± 2.5 (4)	0	2 (2,0)	20	16.4
21p	nr		nr				
22p	nr		nr				
Xp/Yp	nr		nr				
Chr-qarm							
1q	4.3 ± 3.6 (25)	1	3.9 ± 2.0 (5)	0	2 (2,0)	17	12
2q	2.5 ± 2.4 (30)	1	3.6 ± 1.1 (3)	0	3 (3,0)	9	8.6
3q	nd	0	4.1 ± 9.2 (7)	4	1 (0,1)	100	24.9
4q	N/A	N/A	N/A	N/A	N/A	N/A	N/A
5q	4.1 ± 3.3 (34)	0	2.4 ± 0 (1)	0	2 (2,0)	3	14.4
6q	3.3 ± 3.6 (24)	6	nd	nd	0 (0,0)	0	13.8
7q	4.0 ± 8.0 (22)	2	4.7 ± 2.3 (6)	0	8 (7,1)	21	47.3
8q	4.0 ± 4.0 (28)	1	2.1 ± 0 (1)	nd	1 (1,0)	0	12.9
9q	1.3 ± 2.0 (7)	4	nd	0	0 (0,0)	0	4.4
10q	3.6 ± 4.1 (20)	1	7.8 ± 6.8 (2)	0	2 (2,0)	9	13.6
11q	3.0 ± 4.7 (10)	4	nd	0	1 (1,0)	0	13.6
12q	4.9 ± 4.3 (31)	2	nd	0	3 (3,0)	0	13.7
13q	3.4 ± 2.8 (27)	3	nd	0	1 (1,0)	0	9.3
14q	1.7 ± 3.0 (23)	4	nd	0	8 (8,0)	0	14.3
15q	4.8 ± 3.5 (30)	1	nd	0	nd	0	14.3
16q	1.3 ± 1.1 (20)	2	4.4 ± 4.6 (4)	0	4 (4,0)	17	3.5
17q	3.1 ± 4.3 (30)	2	4.8 ± 0 (1)	0	4 (4,0)	3	20.9
18q	3.9 ± 3.3 (31)	1	4.9 ± 3.0 (6)	0	2 (2,0)	16	12.3
19q	5.1 ± 5.4 (31)	0	2.6 ± 2.3 (2)	0	1 (1,0)	6	20.5
20q	1.6 ± 3.2 (12)	3	1.3 ± 1.3 (17)	1	12 (8,4)	59	16.5
21q	4.1 ± 3.8 (28)	0	5.1 ± 2.4 (4)	0	3 (3,0)	11	15.3
22q	INP		INP				
Xq/Yq	1.2 ± 1.9 (16)	3	2.6 ± 1.3 (4)	0	3 (3,0)	20	7.5
Total	780	38	82	5	92 (87,5)	11

**Table 3:** Saos-2 Telomere lengths

	End telomere	End telomere loss	Recombined Ends with ITS	ITS Loss	(TTAGGG) n < 500 bp	% Recombinant Subtelomere Molecules % of total analyzed for subtelomere	Longest Telomere
Chr-parm	Mean Length (kb) ± Std (# telomeres)	(# molecules)	Mean Length (kb) ± Std (# telomeres)	(# molecules)	# molecules (# end, # ITS)		Length (kb)
1p	4.8 ± 8.6 (14)	3	1.6 ± 1.8 (23)	3	5 (3,2)	62	30.5
2p	4.5 ± 3.5 (31)	1	nd	0	1 (1,0)	0	11.0
3p	7.5 ± 5.5 (30)	1	nd	0	1 (1,0)	0	25.5
4p	3.3 ± 2.7 (24)	2	nd	0	0 (0,0)	0	9.9
5p	5.9 ± 5.6 (14)	0	2.6 ± 1.6 (7)	0	0 (0,0)	41	18.3
6p	7.7 ± 7.6 (10)	1	3.1 ± 0 (1)	0	0 (0,0)	10	18.4
7p	N/A	N/A	N/A	N/A	N/A	N/A	N/A
8p	N/A	N/A	N/A	N/A	N/A	N/A	N/A
9p	4.7 ± 4.2 (17)	1	nd	0	0 (0,0)	0	13.6
10p	2.1 ± 1.7 (19)	2	nd	0	2 (2,0)	0	6.7
11p	5.3 ± 5.1 (7)	0	5.0 ± 5.8 (3)	0	0 (0,0)	30	11.8
12p	6.5 ± 4.7 (26)	2	nd	0	0 (0,0)	0	15
13p	nr		nr				
14p	nr		nr				
15p	nr		nr				
16p	INP		INP				
17p	INP		INP				
18p	3.6 ± 3.0 (31)	0	1.1 ± 0 (1)	0	2 (2,0)	3	13.1
19p	4.1 ± 4.0 (30)	1	5.1 ± 3.9 (5)	0	4 (4,0)		13.2
20p	2.0 ± 1.5 (20)	1	nd	0	3 (3,0)	0	4.9
21p	nr		nr				
22p	nr		nr				
Xp/Yp	nr		nr				
Chr-qarm							
1q	4.1 ± 4.6 (25)	3	2.9 ± 2.0 (4)	0	2 (2,0)	3	12.9
2q	N/A	N/A	N/A	N/A	N/A	N/A	N/A
3q	0 ± 0 (1)	1	0.9 ± 0.6 (7)	0	3 (0,3)	88	1.8
4q	0.7 ± 0.9 (3)	1	nd	0	1 (1,0)	0	1.7
5q	5.7 ± 3.6 (27)	0	1.5 ± 0 (1)	0	0 (0,0)	4	12.4
6q	8.9 ± 10.0 (27)	2	14.5 ± 3.7 (5)	0	2 (2,0)	16	50
7q	1.9 ± 1.4 (27)	0	1.3 ± 0 (1)	0	2 (2,0)	4	6.1
8q	1.1 ± 0.7 (15)	1	1.4 ± 0.9 (18)	0	3 (3,0)	55	3.4
9q	N/A	N/A	N/A	N/A	N/A	N/A	N/A
10q	3.0 ± 3.9 (30)	0	0.7 ± 0.9 (2)	0	2 (1,1)		15.9
11q	N/A	N/A	N/A	N/A	N/A	N/A	N/A
12q	N/A	N/A	N/A	N/A	N/A	N/A	N/A
13q	6.4 ± 6.2 (30)	1	7.6 ± 6.2 (5)	0	2 (2,0)	14	23.3
14q	2.3 ± 2.7 (26)	1	nd	0	5 (5,0)	0	6.3
15q	N/A	N/A	N/A	N/A	N/A	N/A	N/A
16q	6.8 ± 17.7 (13)	2	nd	0	0 (0,0)	0	62.8
17q	1.6 ± 1.9 (10)	2	1.7 ± 2.5 (16)	0	4 (0,4)	62	10.1
18q	2.6 ± 3.4 (28)	4	nd	0	5 (5,0)	0	13.1
19q	5.1 ± 5.4 (31)	12	2.6 ± 2.3 (2)	0	1 (1,0)		20.5
20q	N/A	N/A	N/A	N/A	N/A	N/A	N/A
21q	1.3 ± 0.6 (6)	1	1.9 ± 0.7 (22)	0	1 (1,0)	79	3.6
22q	INP		INP				
Xq/Yq	N/A	N/A	N/A	N/A	N/A	N/A	N/A
Total	548	46	118	3	52 (42,10)	19	

*N/A* no data, not enough molecules to measure the telomere length

*INP* inverted nick pair (INP) sites

*nr* no reference sequences or significant gaps in the reference sequence

*nd* Not detected

For all the subtelomeres analyzed, specific examples of linked terminal (TTAGGG) n tract end fragments as well as recombinant end fragments were found. Average subtelomere-specific terminal (TTAGGG) n tract lengths, the ratio of terminal end fragments to recombined end fragments, as well as other telomere-associated structural features varied widely depending upon the specific subtelomere.

**Fig. 1:**
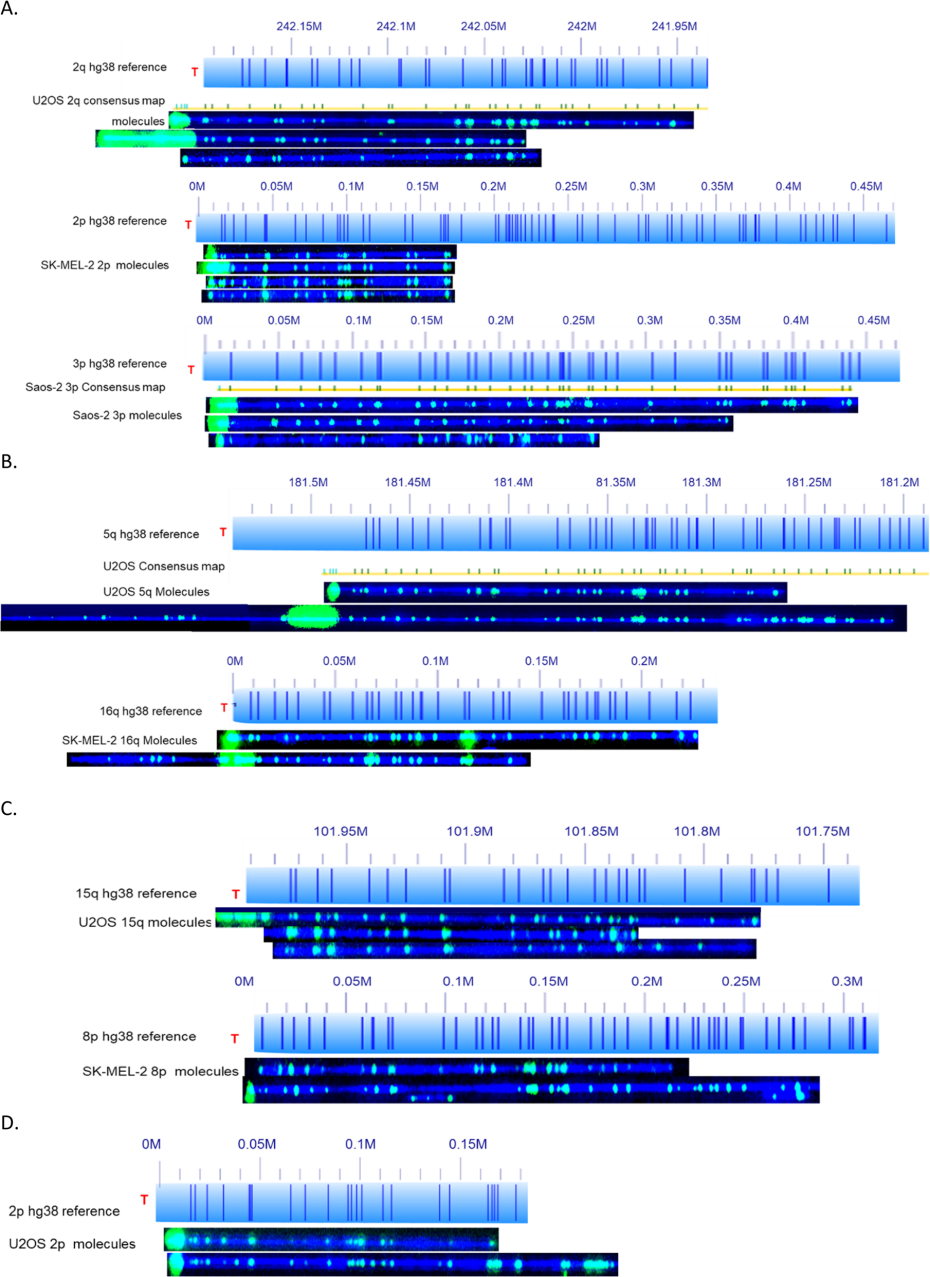
Chromosome arms with low recombination frequency. The hg38 subtelomere reference sequences are shown as light blue bars; the dark blue vertical ticks within these bars indicate in silico Nt.BspQI nick-label sites. Individual single-molecule maps were de novo assembled into the consensus maps (yellow lines), which were then aligned with the hg38 reference (light blue bars). The green lines on the consensus maps are Nt.BspQI (GCTCTTC) sites that align to the corresponding reference site. Those that do not align with the reference are designated with light blue lines. Chromosome arms without a consensus map were mapped to the hg38 reference instead of de novo assembled. The location of the telomere is shown with a red “T”. Representative examples of raw images of single molecules that were imaged are shown below the references. The DNA backbone is blue, and both the labeled subtelomeric Nt.BspQI sites and the telomere (TTAGGG) n tracts are green. **a** Representative data for three chromosome arms (2q U2OS, 2p SK-MEL-2, 3p Saos-2) that have heterogenous end telomere length. **b** A 5q molecule from U2OS with an end telomere and directly below, a 5q end recombined with an unknown genomic fragment with retention of a long internal telomere-like sequence (ITS). 16q molecules from SK-MEL-2, one with recombination and one without. **c** Molecules of 15q from U2OS aligned to hg38; two display end telomere (TTAGGG) n loss. Likewise, molecules from 8p SK-MEL-2, 1 displays telomere loss. **d** U2OS chromosome 2p molecules aligned to hg38 with short end telomeres

The majority of analyzed subtelomeres have mostly terminal (TTAGGG) n ends and less than 50% recombinant ends. The exceptions to this rule were 1q, 3q, 7p, 8q, 11p 18q,19q and 21q of U2OS; 3q and 20q of SK-MEL-2; and 1p, 3q, 8q,17q and 21q of Saos-2, each with with over 50% recombinant telomeres. The longest (TTAGGG) n tracts measured were mostly from terminal telomere ends. In Fig. 1a, examples of individual molecules for 2q (U2OS), 2p (SK-MEL-2) and 3p (Saos-2) are shown with average telomere (TTAGGG) n lengths of 5.5 ± 6.1 kb, 3.1 ± 4.1 kb, and 7.5 ± 5.5 kb respectively. Within the singe molecule datasets corresponding to each subtelomere, (TTAGGG) n tract lengths are highly variable. A good example of this is chromosome arm 2q of U2OS shown in Fig. 1a. One molecule has a telomere length of 17.3 kb compared to another molecule with a telomere length of 0.15 kb. Likewise, differing telomere lengths are also seen in molecules from arm 2p of SK-MEL-2 and arm 3p from Saos-2. The end (TTAGGG) n tract length distribution is highly heterogeneous as indicated by the high standard deviation (Tables 1, 2 and 3). The high variability of end (TTAGGG) n tract lengths observed here is a known characteristic of the ALT mechanism of maintaining telomere lengths [8], and our data show this length heterogeneity extends to all of the specific subtelomeres ending in (TTAGGG) n tracts.

Among these arms with primarily end telomeres, we do see examples of recombinant molecules that often result in internal telomere-like (TTAGGG) n sequence tracts (ITSs; Tables 1, 2 and 3). The ITS length among this group of recombinant molecules is also variable. For example, recombinant telomeres at 1p, 5p, and 11q of U2OS have short ITSs but at 12p there is a high frequency of ITS absence at the recombined telomeres. All 12 recombinant molecules of the 5p end and 5 recombinant molecules of the 11q end have extremely short ITSs of less than 500 bp in length. The three analyzed examples of recombinant 5q ends have longer ITSs with an average telomere length of 6.6 kb ± 3.6; an example is shown in Fig. 1b. By contrast to U2OS, Saos-2 has fewer detected ITS, which concentrated in a few arms.. The SK-MEL-2 has fewest number of ITS.

We unexpectedly observed a very high level of signal-free telomere ends in these three cell lines. A total of 57 out of 781 ends completely lacked detectable (TTAGGG) n end signal detected in the U2OS cell line, 38/818 in SK-MEL-2 and 46/594 in Saos-2. By contrast, we did not observe any signal-free ends in over 5000 single-molecule (TTAGGG) n tract measurements in the senescing IMR90 cell line or the telomerase-positive cancer cell lines UMUC3 and LNCaP [32]. The signal-free ends are distributed unevenly across the arms analyzed for U2OS and SK-MEL-2. Arms 3q, 8q, 14q, and 15q of U2OS have 29 out of 57 signal-free ends (Table 1). 6q, 8p, 8q, 11q, and 14q of SK-MEL-2 have 23 out of 38 signal-free ends (Table 2). But for Saos-2, the signal-free ends are distributed relatively evenly among the arms (Table 3).

Fig. 1c shows several 15q ends of U2OS and 8p of SK-MEL-2 which do not contain detectable (TTAGGG)n. The blue stained DNA backbone extends beyond the first two Nt.BspQI nicking sites without telomere labeling. We scored signal-free ends separately from the (TTAGGG) n lengths acquired from ends with a detectable telomere signal, and did not include them in the average (TTAGGG) n tract length calculations; note that if we had, it would have impacted this metric significantly for some telomeres (e.g., the average telomere length for 15q would have decreased to 3.5 kb from 4.7 kb). Finally, specific subtelomeres (2p, 4p, 12q, 15q) of U2OS have a relatively high number of short (TTAGGG) n tracts amidst a few very long telomeres. An example is shown in Fig. 1d with chromosome arm 2p.

**Fig. 2:**
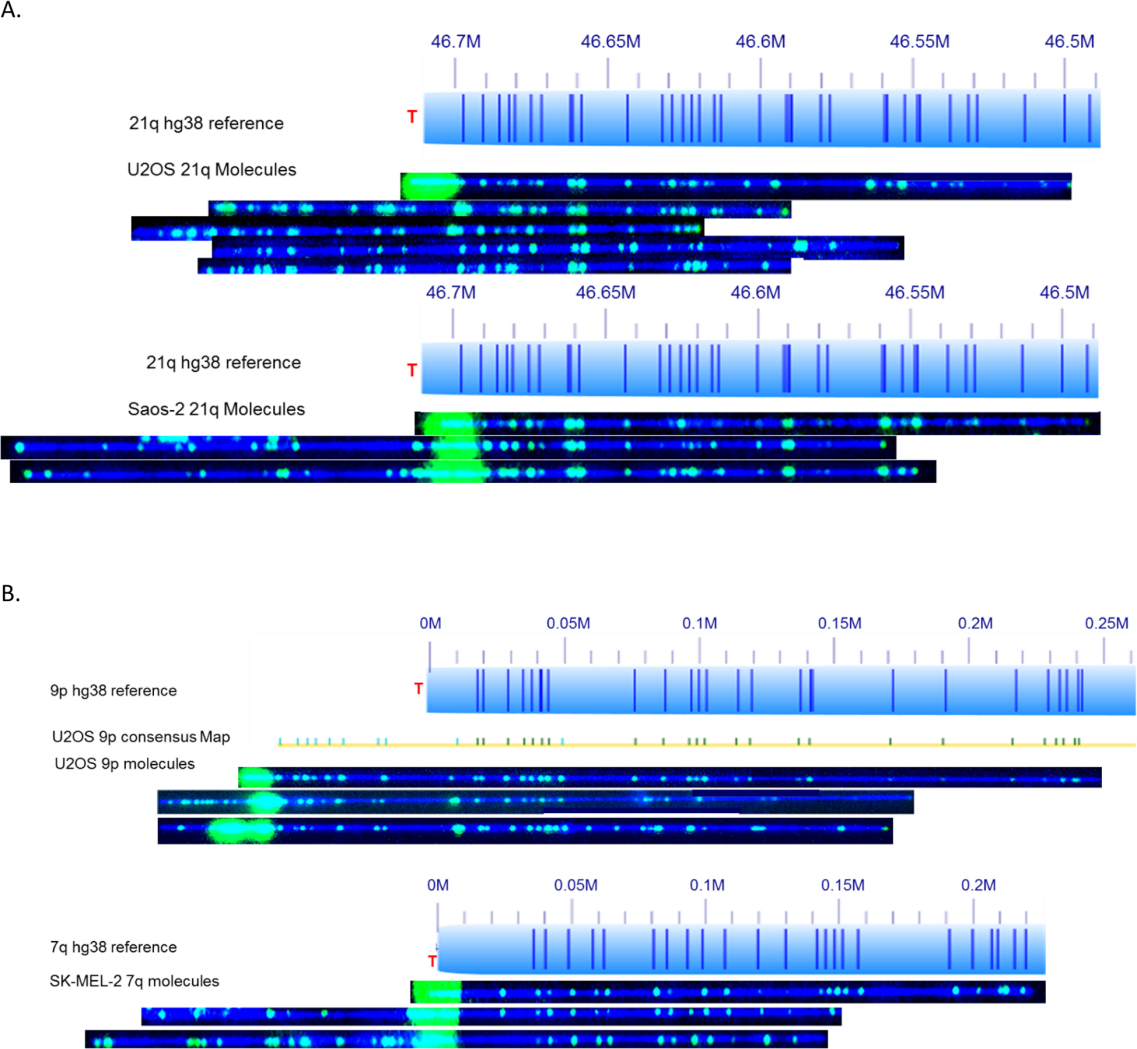
Subtelomeres with a high fraction of recombinant molecules. The hg38 subtelomere reference sequences are shown as light blue bars; the dark blue vertical ticks within these bars indicate in silico Nt.BspQI nick-label sites. The location of the telomere is shown with a red “T”. Representative examples of raw images of single molecules that were imaged are shown below the references. The DNA backbone is blue, and both the labeled subtelomeric Nt.BspQI sites and the telomere (TTAGGG) n tracts are green. **a** Chromosome 21q molecules with a defined pattern in the recombination partner. The top U2OS molecule shows 21q with an end telomere. The second and third U2OS molecules are examples of 21q end recombination with retention of an internal telomere-like sequence (ITS). The bottom two U2OS molecules show the same recombination event but lack a detectible ITS. The top Saos-2 molecule shows 21q with an end telomere. **b** Chromosome 9p molecules from U2OS without a defined pattern in the recombination partner. Chromosome 7q molecules from SK-MEL-2 without a defined pattern in the recombination partner. Individual single-molecule maps were de novo assembled into the consensus maps (yellow lines), which were then aligned with the hg38 reference (light blue bars). The green lines on the consensus maps are Nt.BspQI (GCTCTTC) sites that align to the corresponding reference site. Those that do not align with the reference are designated with light blue lines. The top U2OS molecule shows a structural variant of the 9p end with an end telomere. The bottom two U2OS molecules show the same structural variant of the 9p end following recombination resulting in two distinct partner fragments, with retention of ITSs in both cases. Likewise the top 7q SK-MEL-2 molecule has an end telomere while the bottom two show recombinants with ITS retention

Recombinant telomeres and ITSs were seen in three ALT positive cell lines. U2OS has the highest fraction of recombinant telomeres at the average of 24%; Saos-2 has 19%; SK-MEL-2 has the lowest fraction at only 11%. The 1q, 3q, 6p, 7p, 8q, 9p, 11p, 18q, 19q, and 21q arms of U2OS have the highest fraction of recombinant telomeres. 1q, 3q, 9p, 18q, and 21q are shown to have high fractions of recombinant telomeres in SK-MEL-2. In Saos-2, 1p, 3q, 8q, 17q and 21q each have over 50% recombinant telomeres. The recombination partner DNA fragment for most of these subtelomeres typically shows a defined stable pattern (Fig. 2a). The 21q subtelomere of U2OS shown in Fig. 2a has a combination of molecules with end telomeres, recombination with retention of ITS, and recombination without retention of ITS. The 21q arm telomeres retained ITS length (1.9 ± 4.0 kb) for recombinant molecules is significantly shorter than the end telomere length (5.3 ± 4.7 kb). The 21q arm of Saos-2 is also highly recombined with similar average telomere length (1.9 kb ± 0.7) in comparison to the end telomere length (1.3 kb ± 0.6). The recombined patterns of 21q are different between U2OS and Saos-2. Figure 2b shows that 9p of U2OS has a defined recombination pattern, while 7q of SK-MEL-2 lack defined patterns.).

Examples of very short ITSs at recombinant telomeres are 1q, 6p, 7p, 11p, and 18q of U2OS, 20q of SK-MEL-2 and 3q of Saos-2, all with multiple detected internal telomeres averaging between 0.4 kb and about 1.3 kb. Overall, recombinant molecules of U2OS have the highest fraction of ITS loss (108 molecules with ITS loss compared to 182 molecules with ITS) among these three cell lines. Chromosome 3q ends of U2OS had no detectable (TTAGGG) n tracts at all, with 7 end molecules and 16 recombinant molecules all lacking telomere signal (Table 1). On the other hand, SK-MEL-2 and Saos-2 have lower fractions of ITS loss (5 vs. 82 of SK-MEL-2, and 3 vs. 118 of Saos-2) compared to U2OS.

**Fig. 3:**
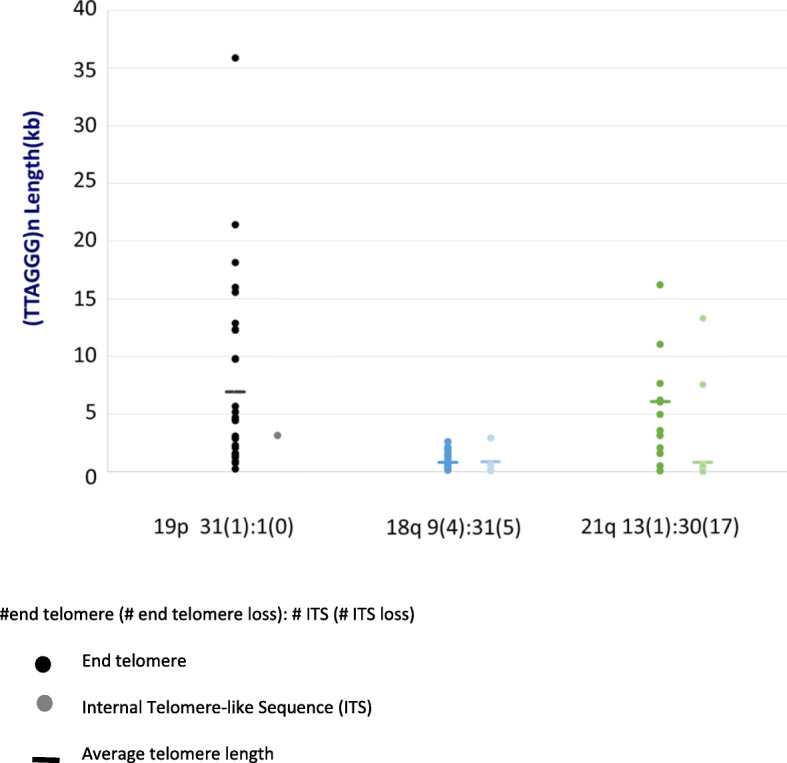
End telomere lengths and retained ITSs at specific recombined telomeres. Telomere (TTAGGG) n tract length results for 19p, 18q, and 21q from U2OS. Each single molecule telomere length measurement is represented by a dot and the average telomere length for each chromosome arm is shown as a horizontal line. For each chromosome arm, the left group of dots is from telomere end molecules. The total number of molecules is reported below with the number of molecules with complete telomere tract loss in parentheses. The right group of dots is from molecules with recombined telomere ends. The total number of recombined molecules is reported below with the number of recombined end molecules lacking retention of a detectable (TTAGGG) n tract indicated in parentheses

Figure 3 shows (TTAGGG) n telomere tract length distributions for 19p, 18q, and 21q of U2OS, illustrating the variability of this parameter depending upon the subtelomere involved. Chromosome 19p ends are comprised almost exclusively of molecules with (TTAGGG) n tracts, with long and heterogeneous tract lengths having an overall average of 7.2 kb. Chromosome 18q ends are mostly recombinant; the limited number of molecules with end telomeres have very short (TTAGGG) n tracts (0.9 kb ± 1.2), with 4 end molecules lacking any signal. The ITSs associated with recombinant 18q molecules are similarly short (0.9 kb ± 0.7) or absent (5 molecules). The 21q subtelomere molecules have a broad range of heterogeneously sized (TTAGGG) n tracts on their ends with mostly very short or absent ITSs in recombinant telomeres.

**Fig. 4:**
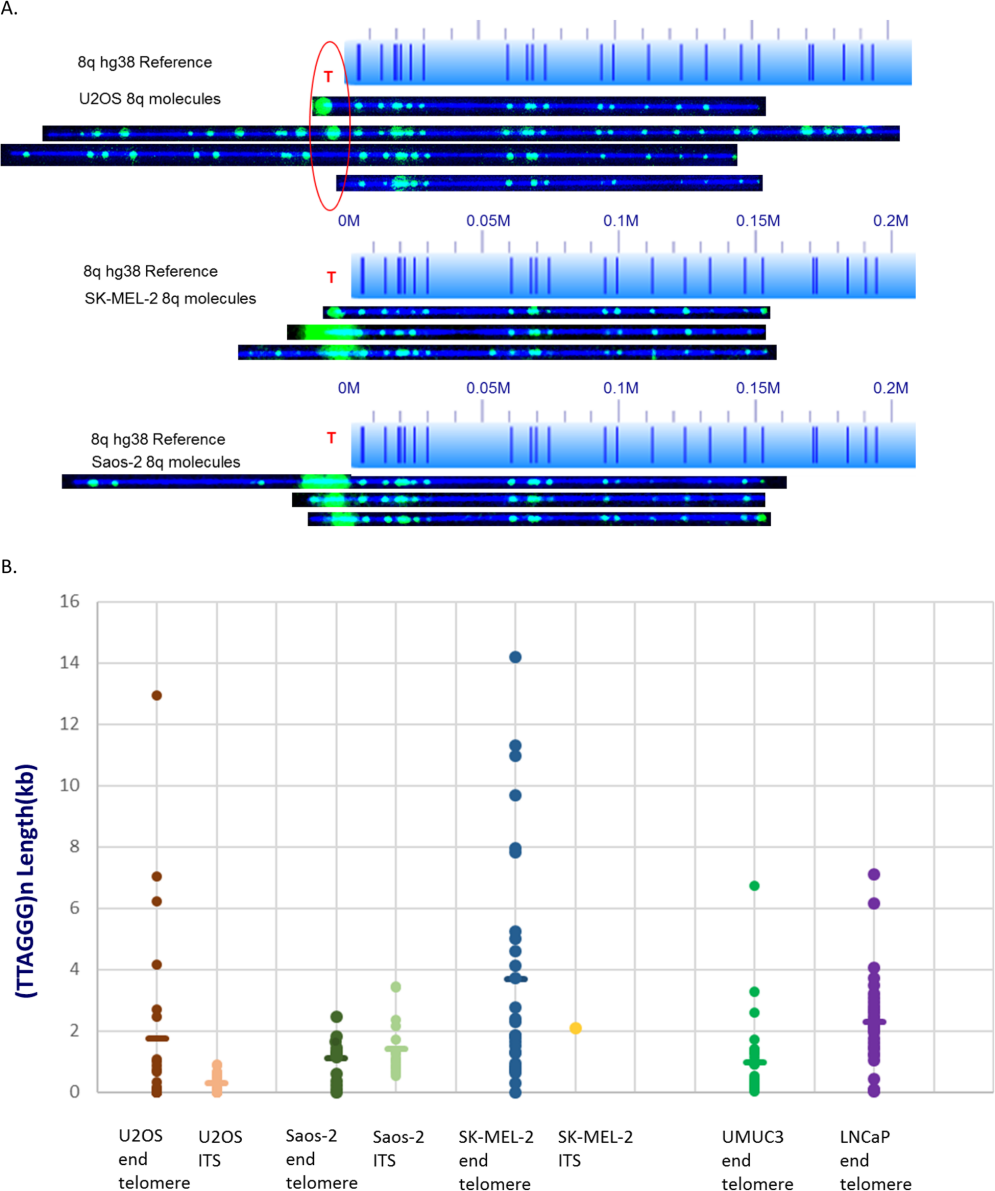
Comparison of 8q for U2OS, UMUC3, and LNCaP. **a** The hg38 subtelomere reference sequence for 8q is shown as a light blue bar; the dark blue vertical ticks within this bar indicate in silico Nt.BspQI nick-label sites. The location of the telomere is shown with a red “T”. Representative examples of raw images of single molecules that were imaged are shown below the reference for each of the cell lines. The DNA backbone is blue, and both the labeled subtelomeric Nt.BspQI sites and the telomere (TTAGGG) n tracts are green. The top U2OS molecule has an end telomere with a (TTAGGG) n tract, the second has undergone recombination with retention of an ITS, telomere, the third has undergone recombination without retention of an ITS, and the bottom molecule has an end telomere loss. The Saos-2 molecules retain their ITS., but SK-MEL-2 has only one ITS. **b** Comparison of U2OS, Saos-2 and SK-MEL-2 end telomeres and internal telomere length with UMUC3 and LNCaP end telomere length. Each single molecule telomere length measurement is represented by a dot and the average telomere length for each chromosome arm is shown as a horizontal line. UMUC3 and LNCap don’t have any ITS due to recombination

From our previous single-molecule telomere length analyses of senescing primary IMR90 fibroblasts and cancer cell lines UMUC3 and LNCaP, we found that the distribution of very short single telomeres was biased with an unusually high fraction of very short telomeres at 8q for all three cell lines, and also at 14q for IMR90 [32]. We therefore looked for unusual (TTAGGG) n length distributions at these telomeres in the U2OS, SK-MEL-2, and Saos-2 cancer cell lines. The typical single molecule images with telomere tracts are shown in Fig. 4a. For U2OS, 8q has relatively short (TTAGGG) n tracts at 8q with the average length of 1.8 kb as shown in Fig. 4b. The (TTAGGG) n tracts are highly variable, ranging from 6 molecules featuring telomere loss, 6 ends with detectable (TTAGGG) n lengths less than 500 bp, and 11 end-molecules having a heterogeneous size distribution from 500 bp to 12.9 kb, with only 4 end molecules having (TTAGGG) n tracts greater than 4 kb in size (Table 1;Fig. 4b). For U2OS, 8q also has a high fraction of recombinant ends, with 7 out of 26 of these molecules lacking ITSs and the remaining 19 recombinant molecules averaging 0.4 kb-sized ITSs (Table 1, Fig. 4b). Saos-2 8q behaves similarly to U2OS 8q. Besides very short telomere ends (1.1 kb average telomere length), Saos-2 8q also has a high fraction of recombinant ends, but Saos-2 8q has fewer end telomere and ITS loss. SK-MEL-2 8q seems to have a different profile compared to U2OS and Saos-2 8q. It not only has longer end telomere (4 kb average length), but also lacks recombinant molecules.

Overall, the end telomere lengths of U2OS, SK-MEL-2, and Saos-2 are highly variable ranging from undetectable to extremely long (Tables 1, 2, and 3) in comparison to UMUC3 and LNCaP which are documented to have relatively uniform and short telomere length distributions [37, 38]. When looking specifically at 8q ends, mean (TTAGGG) n tract lengths are similar in the ALT-positive U2OS, SK-MEL-2 and Saos-2 cancer cell lines and telomerase-positive cancer cell lines. Few very long telomeres are found at U2OS and SK-MEL-2 8q distinguishing the ALT positive from the telomerase positive cell lines at this telomere (Fig. 4b). On the other hand, Saos-2 completely lacks long telomeres with lower heterogeneity.

Similarly, 14q was enriched for short telomeres in IMR90 [32]; in U2OS, 14q end-molecules had the highest fraction of signal-free ends (10/16 molecules) and all 4 recombined 14q ends lacked ITSs (Table 1). The average end telomere length of U2OS is at 1.6 kb. SK-MEL-2 14q also has short average telomere length of 1.7 kb with only 4 molecules having end telomere loss. Saos-2 has only one end telomere loss at 14q with relatively longer average telomere length of 2.3 kb. The overall end telomere length and heterogeneity is higher than in telomerase-positive cancer cell lines. At some specific ends lengths appear to be very similar, perhaps implying a level of active cis control of the shortest telomeres in both pre- and post-immortalization cells, irrespective of TMM.

**Fig. 5:**
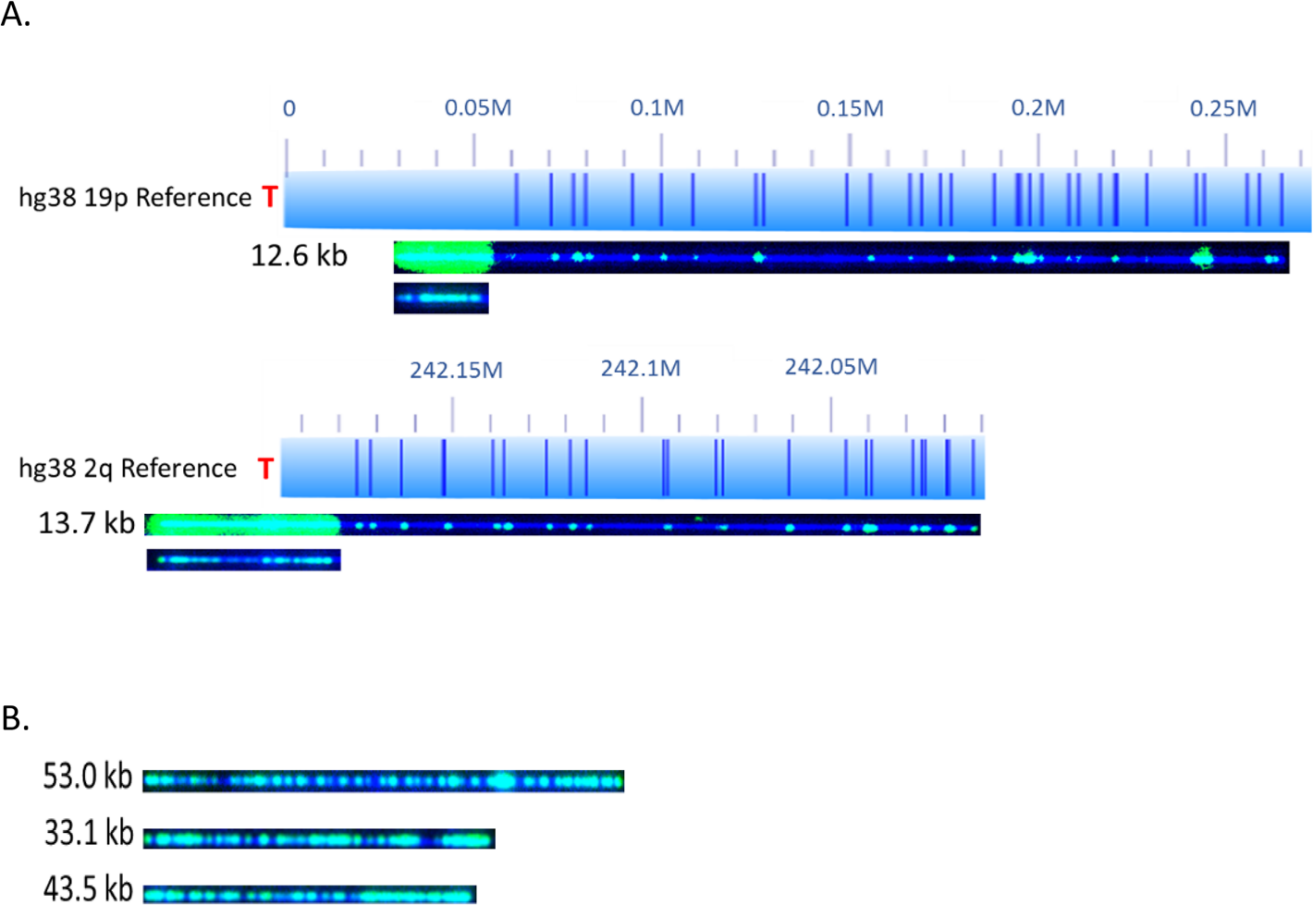
Punctate and Extrachromosomal Telomeres. Representative examples of raw images with the DNA backbone blue, and both the labeled subtelomeric Nt.BspQI sites and the telomere (TTAGGG) n tracts are green. **a** Examples of end telomeres with punctate (TTAGGG) n tracts. The intensity of these telomere tracts is decreased and shown below the full molecule. **b** Examples of Extrachromosomal telomeres, many of which display punctate (TTAGGG) n tracts

Telomeres with punctate (TTAGGG) n labeling patterns were observed at many chromosome ends (approximately 65%) in all three ALT cell lines at nearly all long extrachromosomal telomere repeat (ECTR) DNA fragments (89%) using our single-molecule analysis methods (Fig. 5). This punctate labeling feature was not observed on any telomeres from IMR90 fibroblasts or from telomerase-positive cell lines [32]. The punctate feature of the labeling suggests stretches of nontelomeric DNA sequence and/or variant (TTAGGG) n -like repeat DNA interspersed with pure (TTAGGG) n in these telomere tracts, as described previously [20, 39]. While ECTR DNA including c-circles, t-circles, and small linear (TTAGGG) n fragments have long been known to be closely associated with ALT-positive cells, with the small linear ECTRs specifically found to be closely associated with ALT-associated PML bodies [31, 40], it was a surprise to discover the very large linear ECTRs using our single-molecule analysis method (Fig. 5b). Large linear ECTRs comprised 40% of the total telomere signal in U2OS; the average telomere length for these ECTRs was 11.0 kb with the longest measuring 50.4 kb. The existence and abundance of large linear ECTRs is a new insight with potentially important ramifications for mechanisms of DNA repair and telomere maintenance at ALT telomeres, since they can potentially serve as template molecules for long-tract break-induced repair (BIR) to generate long stretches of new telomeric DNA tracts at damaged telomeres. The same patterns of long linear ECTRs are also observed in SK-MEL-2 and Saos-2 cell lines.

## Discussion

Our general global observation of long end telomere tracts and telomere length heterogeneity was anticipated from prior studies of ALT cells [8]. However, the resolution and subtelomere specificity of our single-molecule analysis revealed many unanticipated details. First, average telomere tract length and heterogeneity level of end-telomeres varied dramatically depending on the linked subtelomere and the cell line, with a few subtelomeres associated with mostly very short end telomeres, ends lacking any telomere signal (“signal-free ends”), and fused telomeres. Overall, there were a surprising number of signal-free ends (8%) in U2OS cells and SK-MEL-2 cell lines (8%), and over half of these were associated with just a few subtelomeres. On the other hand, the signal-free ends are more evenly distributed across subtelomeres in Saos-2 cells (5%). By contrast, there were no signal-free ends found in telomerase negative senescing IMR90 fibroblasts, telomerase-positive cancer cell lines (UMUC3, LNCaP) and lymphoblastoid cell lines [32]. Second, there was a large fraction of recombinant telomeres with variable tract lengths of internal telomere-like sequence (ITS) that were retained at the fusion junctions. As with the end-telomere characteristics, features of the recombinant telomere molecules (including the percentage of the specific subtelomere molecules involved, and the relative size and retention of the ITS at the fusion site) varied depending upon the linked subtelomere and the cell line. Third, very large linear ECTR molecules were present in all three ALT positive cell lines; most of these (along with many large end-telomere tracts) displayed punctate CRISPR/Cas9-dependent (TTAGGG) n labeling patterns indicative of interspersion of stretches of non-canonical telomere repeats.

Ploidy changes, losses and gains of whole chromosomes, and especially structural chromosome aberrations characteristic of ALT cells are expected to be reflected in our results. Previous karyotypic analysis of ALT cell lines [41, 42] revealed extensive genome rearrangement including hyper-triploid and higher chromosome numbers as well as frequent non-reciprocal translocations, deletions, and complex chromosome rearrangements. These types of genome rearrangements are believed to be primarily a consequence of telomere dysfunction [43, 44] and likely occurred in these cells prior to immortalization by ALT, but there is also evidence of ongoing, albeit lower frequency, genome instability in immortalized ALT cells [41, 42]. In a study which included U2OS and Saos-2 cell lines, clonal structural chromosomal anomalies in the karyotypes of 9 ALT cell lines was 3.7- fold higher than in telomerase-positive cancer cell lines [42]. In the same study, approximately 45% of telomere signals localized to structurally aberrant chromosome arms or to marker chromosomes. The low resolution of the metaphase mapping precluded distinguishing telomere capture events (which would stabilize the arm by translocation of a large telomere-terminal chromosome fragment, including the native subtelomere) from telomere healing (direct addition of (TTAGGG) n sequences to a broken chromosome). Internal telomere-like sequences (expected to result from the fusion of dysfunctional telomeres with either other chromosome ends or with non-telomeric genome sites; and also by other possible mechanisms [45]) were previously detected by metaphase FISH in the karyotypes of many ALT cell lines, including U2OS and Saos-2 cells [42].

The types of large single-telomere-associated molecules we analyzed can be explained in part in the context of these types of clonal structural and numerical chromosome changes in ALT. Molecules with end telomeres must arise from the original chromosome ends, from telomere capture fragments translocated onto broken chromosomes, or from internal chromosome breaks healed by direct addition of (TTAGGG) n tracts. In our dataset, only 70% of end-telomeres were linked to recognizable subtelomeres (data not shown), suggesting that a sizable fraction of end-telomeres may result from telomere “healing” onto non-telomeric sites of chromosome breaks. The molecular correlates of ITS-generating chromosome aberrations in our dataset are expected to be recombinant telomere molecules with retained ITSs sufficiently large enough for detection by metaphase FISH. Recombinant telomere molecules with defined partner molecules are expected to be clonal descendants of telomere fusion events; those with multiple partners must be subject to ongoing breakage and re-joining events, perhaps similar in principle to yeast breakage-fusion-bridge cycle events in which successive breakage events preferentially localize to the original telomere fusion sites and to pericentromeres [46, 47].

Recombinant telomeres linked to specific subtelomeres were surprisingly fluid in their retention and tract length variation of ITSs at the fusion site. For example, recombinant molecules linked to 21q in U2OS were associated with the same partner genomic DNA fragment, yet single molecules were found with the ITS retained, the ITS lost, and ITSs with discrete sizes retained (Table 1, Fig. 2a). If the entirety of these molecules are derived from a single clonal precursor, then this result indicates ongoing evolution of the junction site (ITS length change, ITS loss, or ITS gain) during U2OS propagation. For 3q in U2OS, all examples of recombinant telomeres lack retention of ITS; curiously, all examples of 3q-linked ends also lack telomere signal (Table 1), perhaps suggesting the co-existence of precursor 3q ends lacking telomeres and recombinant 3q in U2OS cultures. By contrast, in instances where recombinant telomere molecules from the same subtelomere have multiple distinct partner fragments, the simplest explanation is that there have been independent recombination events involving the same subtelomere locus suggesting the possibility that multiple breaks in breakage-fusion-bridge cycles have occurred within the telomere fusion sites, as has been observed in Sacharomyces cerevisiae [46, 47].

Subtelomeres we detected using our optical mapping method in six other cell lines but which were rarely found or not found in the dataset (4q, 8p, 10p, 10q, and XqYq of U2OS; 5p, and 11p in SK-MEL-2; 2q, 7p, 8p, 9q, 11q, 12q, 15q, 20q and XqYq in Saos-2) may have been associated with cycles of breakage and recombination that resulted in loss of their original sequence organization. In this context, it is notable that two of these subtelomeres, 4q and 10q, contain large tandem arrays of 3.3 kb repeats (D4Z4) that are expected to de-stabilize these subtelomeres in an environment of enhanced homologous recombination. Alternatively, some of these missing or rearranged subtelomeres may have suffered homozygous deletion in an early stage of the crisis/immortalization process and were simply missing in all subsequent generations of the cell line.

These results demonstrate the power and utility of our single-molecule telomere analysis method for analyzing ALT mechanisms providing new insight into structural events associated with ALT telomeres and demonstrating subtelomere specific differences in these events. The path is now open to a more detailed and directed analyses of hypothesized mechanisms involved in ALT. For example, subtelomere specificity of telomere length regulation and stability may relate in part to TERRA transcription [22–25]. TERRA is critical to telomere length regulation and stability, transcribed from subtelomeric promoters, and differentially expressed at single telomeres making it an attractive candidate for modulating single telomere specific features of ALT. There are believed to be, as yet unknown, non-canonical recombination and repair pathways involved in ALT [21, 31] and our method of structural analysis could be key in helping to sort these out. Different ALT conditions will likely produce different categories and frequencies of telomere structural events, and the methods demonstrated here will provide a unique tool to track these at the single-molecule and subtelomere-specific level.

## Conclusions

By measuring (TTAGGG) n tract lengths from the same large DNA molecules that were optically mapped, we simultaneously analyzed telomere length dynamics and subtelomere-linked structural changes at a large number of specific subtelomeric loci in the ALT-positive cell lines U2OS, SK-MEL-2 and Saos-2. Our results revealed loci-specific ALT telomere features including terminal (TTAGGG) n tract lengths and heterogeneity levels, the frequencies of signal-free ends, the frequencies of recombinant telomeres and the presence/absence and size of retained internal telomere-like sequences (ITSs) at these telomere fusion junctions, and the presence of large linear extrachromosomal telomere repeat (ECTR) DNA molecules. All of these telomere-linked features are dramatically distinct from previously characterized somatic cell (IMR90) and telomerase-positive cancer cell (UMUC3 and LNCaP) genomes we characterized previously using the same methodology (32). These results reveal in molecular detail the structural consequences of telomere damage, repair and recombination mechanisms in human ALT and how they vary in different ALT genomic backgrounds.

## Methods

### Cell preparation

The U-2 OS (ATTC® HTB-96™) Saos-2 (ATCC® HTB-85™) and SK-MEL-2 (ATCC® HTB-68™) cell lines were acquired from American Type Culture Collection (ATCC). The U2OS cell line was cultured in McCoy’s 5a Medium supplemented with 10% Fetal Bovine Serum (FBS) (Corning). The Saos-2 cell line was cultured in McCoy’s 5a Medium supplemented with 15% FBS. The SK-MEL-2 cell line was cultured in Eagle’s Minimum Essential Medium supplemented with 10% FBS. The cells were passaged using 0.25% trypsin-EDTA (Gibco).

**High molecular weight DNA extraction, guide RNA preparation and the two color DNA labeling scheme:** These procedures are followed exactly as described in McCaffrey et al. [32]**.**

### DNA loading and imaging

After nick-labeling, the samples were treated with Protease and IrysPrep Stop Solution (BioNano Genomics) [32]. The labeled DNA was stained with YOYO-1 (Invitrogen), and was loaded into the nanochannels following the established protocol [33]. We generally collected 60x coverage (180Gb) data to obtain 30 molecules containing the telomeres for each chromosome. The image analysis was done following the established procedure [32].

### De novo genome map assembly

Single DNA molecules were assembled de novo into consensus maps or aligned to hg38 reference using software tools developed at BioNano Genomics, specifically Refaligner and Assembler [48]. The de novo assembly procedure is the same as described in McCaffrey et al. [32].

### Telomere length analysis

The telomere labels were found to be the extra labels at the end of molecules or in the middle of recombinant molecules. The length of the telomeres was inferred from its fluorescent label intensity following the established protocols [32].

## Data Availability

The BioNano whole-genome mapping data from this study have been submitted to the NCBI BioProject (https://www.ncbi.nlm.nih.gov/bioproject/396850) under accession number PRJNA396850. The BioNano supplemental data at this accession refer to SKMEL (SUPPF_0000003660), Saos2 (SUPPF_0000003661) and U2OS (SUPPF_0000003665). The hg38 reference used was from the Genome Reference Consortium, the December 2013 version GRCh38/hg38 GCA_000001405.15, and downloaded from the UCSC Genome Browser, at http://hgdownload.soe.ucsc.edu/goldenPath/hg38/chromosomes/.

## Abbreviations

ALT: Alternative Lengthening of Telomeres
BIR: Break-induced repair
DDR: DNA Damage Response
ECTR: Extrachromosomal telomere repeat
FISH: Fluorescence in situ hybridization
ITSs: Internal telomere-like sequences
PML: Promyelocytic leukemia
